# Supplementary material for: Changes in Estimating the Wild Boar Carcasses Sampling Effort: Applying the EFSA ASF Exit Strategy by Means of the WBC-Counter Tool
Source: Viruses. 2022 Jun 28;14(7):1424. doi: 10.3390/v14071424 (PMC9319840; doi:10.3390/v14071424)
Supplement: Supplementary file 1 [file viruses-14-01424-s001.zip › viruses-1742158-supplementary.pdf]

Table S1. Data on carcasses used to implement the Sardinian Exit Strategy. Data are reported by Hunting management Units with associated starting data of the Exit Strategy, data of carcasses founding, location of founding (municipality), and related phase of screening or confirmation.

Hunting Management Unit *	Date of Finding	Municipality	Phase
Goceano-Gallura (29/11/2020)	13/02/2021	Bono	screening
	23/07/2021	Olbia	screening
	02/08/2021	Olbia	screening
	22/08/2021	Alà dei Sardi	confirmatory
	04/09/2021	Padru	confirmatory
	20/10/2021	Orotelli	confirmatory
	12/02/2022	Bono	confirmatory
	10/02/2022	Illorai	confirmatory
Nuoro-Baronia (06/01/2020)	29/01/2020	Oliena	screening
	10/08/2020	Nuoro	confirmatory
	09/10/2020	Bitti	confirmatory
	19/03/2021	Golfo Aranci	confirmatory
	12/04/2021	Nuoro	confirmatory
	15/09/2021	Nuoro	confirmatory
	30/10/2021	Nuoro	confirmatory
	12/11/2022	Bitti	confirmatory
	15/11/2021	Golfo Aranci	confirmatory
	19/11/2021	Posada	confirmatory
Gennargentu-Ogliastra (17/01/2021) Total	20/05/2021	Aritzo	screening
	02/08/2021	Nurri	screening
	17/08/2021	Seui	screening
	30/08/2021	Baunei	confirmatory
	08/09/2021	Nurri	confirmatory
	20/10/2021	Sadali	confirmatory
	29/11/2021	Olzai	confirmatory
	16/12/2021	Talana	confirmatory
	18/03/2022	Triei	confirmatory
	26/04/2022	Gadoni	confirmatory

* Data are reported as name of the area and starting date (dd/mm/yyyy).
